# Chemical genetic approach using β-rubromycin reveals that a RIO kinase-like protein is involved in morphological development in *Phytophthora infestans*

**DOI:** 10.1038/s41598-020-79326-7

**Published:** 2020-12-18

**Authors:** Shuji Tani, Naotaka Nishio, Kenji Kai, Daisuke Hagiwara, Yoshiyuki Ogata, Motoaki Tojo, Jun-ichi Sumitani, Howard S. Judelson, Takashi Kawaguchi

**Affiliations:** 1 Graduate School of Life and Environmental Sciences, Osaka Prefecture University, Osaka, 599-8531 Japan; 2 Medical Mycology Research Center, Chiba University, Chiba, Japan; 3 Faculty of Life and Environmental Sciences, University of Tsukuba, Tsukuba, Ibaraki Japan; 4 Department of Microbiology and Plant Pathology, University of California, Riverside, Riverside, CA USA

**Keywords:** Genetics, Microbiology, Molecular biology

## Abstract

To characterize the molecular mechanisms underlying life-stage transitions in *Phytophthora infestans*, we initiated a chemical genetics approach by screening for a stage-specific inhibitor of morphological development from microbial culture extracts prepared mostly from actinomycetes from soil in Japan. Of the more than 700 extracts, one consistently inhibited *Ph. infestans* cyst germination. Purification and identification of the active compound by ESI–MS, ^1^H-NMR, and ^13^C-NMR identified β-rubromycin as the inhibitor of cyst germination (IC_50_ = 19.8 μg/L); β-rubromycin did not inhibit growth on rye media, sporangium formation, zoospore release, cyst formation, or appressorium formation in *Ph. infestans*. Further analyses revealed that β-rubromycin inhibited the germination of cysts and oospores in *Pythium aphanidermatum*. A chemical genetic approach revealed that β-rubromycin stimulated the expression of RIO kinase-like gene (PITG_04584) by 60-fold in *Ph. infestans*. Genetic analyses revealed that PITG_04584, which lacks close non-oomycete relatives, was involved in zoosporogenesis, cyst germination, and appressorium formation in *Ph. infestans*. These data imply that further functional analyses of PITG_04584 may contribute to new methods to suppress diseases caused by oomycetes.

## Introduction

Oomycetes, which are classified as Stramenopiles, include many economically important eukaryotic plant pathogens. In particular, species in the genera *Phytophthora* and *Pythium* are among the most destructive pathogens, affecting crops, shrubs, and trees on a global scale^1^. For example, *Phytophthora cinnamomi* has a very broad host range, with approximately 5000 susceptible species identified^2^. *Pythium aphanidermatum* also has a wide host range, causing damping off, especially in vegetable crops^3^. Other species have more narrow host ranges, such as the historically and economically important *Phytophthora infestans* which infects Solanaceae species and causes late blight of potato and tomato. One study estimates the annual cost of potato late blight at $10 billion dollars, with chemical control representing 10 to 20% of the total cost of production^4^. Various chemical treatments have been developed to control plant diseases caused by oomycetes^5^. However, the emergence of strains resistant to anti-oomycete agents is problematic^6^. Consequently, new chemicals with different modes of action must be developed to control oomycete diseases. To accelerate the development of new anti-oomycete agents, the molecular mechanisms controlling oomycete morphological development and plant infection should be elucidated.

*Phytophthora infestans* produces sporangia, which undergo temperature-induced morphological changes. For example, placing sporangia in warm water (e.g., > 20 °C) for more than 1 h induces direct germination. In contrast, placing sporangia in cool water (e.g., < 12 °C) for more than 1 h stimulates cytoplasmic cleavage and the release of six or more biflagellated zoospores^7^. The resulting zoospores swim through the water, and then form walled cysts on a plant surface, losing their flagella. The cysts germinate to form appressoria that breach the host epidermis. Therefore, the asexual life cycle is important for most natural infections^8^.

Zoospore release is a rapid event that occurs in response to a complex calcium signaling pathway^9,10^. Tani et al. suggested that exposure to cold conditions increases membrane rigidity in sporangia, which activates calcium signaling pathways that drive zoosporogenesis and the transcription of cold-responsive genes^7^. Major transcriptional changes occur during each stage of spore development and germination^11^. Actinomycin D and cycloheximide do not block zoospore release but do block cyst germination, implying that new protein synthesis is required for infection^12^. However, details regarding the molecular mechanisms underlying these developmental transitions remain unclear.

Here we describe the use of a chemical genetic method to study morphological development. We first screened actinomycetes for chemicals that inhibit development since actinomycetes are known as prolific producers of natural products with a wide range of biological activities^13^. This identified β-rubromycin as an inhibitor of *Ph. infestans* cyst germination and hyphal elongation. β-rubromycin was also found to inhibit cyst and oospore germination in *Py. aphanidermatum*. We also discovered that a RIO kinase-like gene of *Ph. infestans,* PITG_04584, was up-regulated by β-rubromycin, and used gene silencing to show that the kinase is involved in multiple development stages.

## Results

### Screening for actinomycetes producing inhibitors of *Ph. infestans* cyst germination

Actinomycetes in soil samples from Japan were isolated on modified humic acid-vitamin (HVG) medium. Compounds from the approximately 700 isolated microorganisms were then screened for their abilities to inhibit zoospore release, cyst formation, and cyst germination in *Ph. infestans*. The microorganisms were grown in liquid culture medium A at 30 °C for 5 d with shaking. Samples comprising 50% acetone (final concentration) were prepared by incubating the culture broth with an equal amount of acetone at 4 °C overnight. After centrifugation, the cell-free extracts were subjected to the assay. The samples were added to 1 × 10^3^ *Ph. infestans* sporangia, incubated at 10 °C for 18 h, and examined with a stereo microscope. While many of the approximately 700 test samples inhibited zoospore release and cyst formation, two samples inhibited cyst germination, but no effect was observed on zoospore release and cyst formation. The sequential dilution of two samples confirmed that only one sample, no. 750, reproducibly inhibited cyst germination in a dose-dependent manner (Supplementary Fig. S1). We then focused on the no. 750 sample for further analysis.

The 16S rRNA gene sequence of the no. 750 strain was identical to that of *Streptomyces massasporeus* strain NBRC 12796. Thus, our isolate was designated as *Streptomyces* sp. no. 750.

### Purification and identification of the inhibitor of *Ph. infestans* cyst germination

To produce large amounts of the active compound from *Streptomyces* sp. no. 750, we optimized the culture conditions by changing the nitrogen and carbon sources (medium A to F, Supplementary Table S1) and the culture duration. Because the 50% acetone extracts from the 5-day cultures in medium E consistently inhibited cyst germination (data not shown), we grew *Streptomyces* sp. no. 750 in culture medium E for 5 d. The only difference between media A and E is the presence of the Pridham-Godleave solution in medium E, implying that trace elements were critical for enhancing the production of the cyst germination inhibitor.

**Figure 1:**
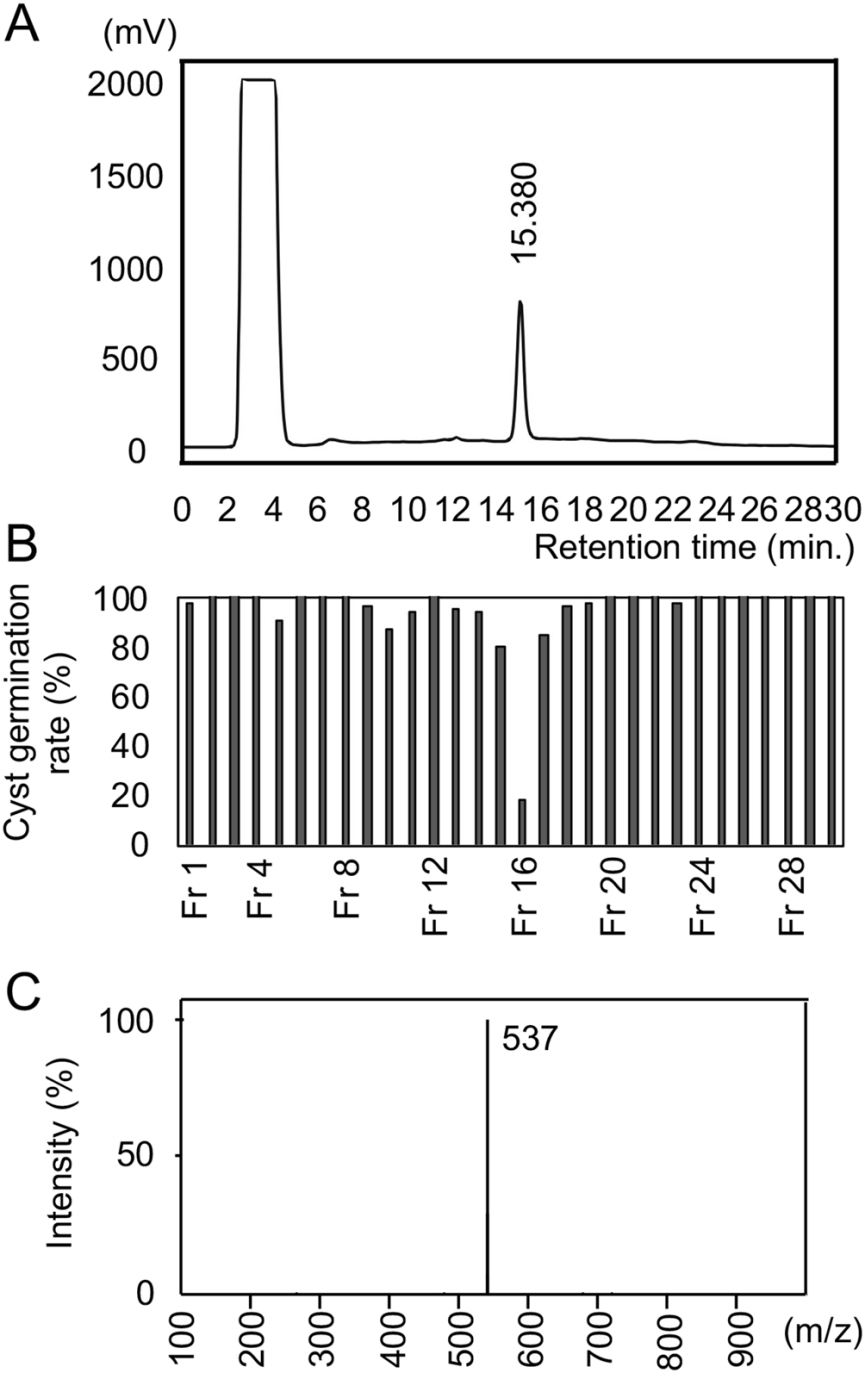
Activity and LC–MS data of purified sample A. (**A**) HPLC profile indicating that the bioactive fraction was at 15.38 min. (**B**) Effect of fractions on *Ph. infestans* cyst germination, showing that the peak from panel A contains the inhibitor. The cyst germination rate under the control condition was set as 100%. (**C**) LC–MS data of Sample A purified from the isolated strain, *Streptomyces* sp. no. 750 (*m*/*z* = 0–1000).

The purification was started from 10.76 g of dried crude extract, which was extracted with ethyl acetate from a 76.4-L culture supernatant followed by evaporation. The ethyl acetate fraction was chromatographed on Wakogel C-200 and Inertsil ODS-3 columns. The germination-inhibiting sample eluted as a single peak with a retention time of 15.380 min, and was dried to yield 2.9 mg of red powder (sample A) (Fig. 1A, B).

The ESI–MS data for sample A revealed a molecular ion [M + H]^+^ at *m/z* 537 (Fig. 1C). A search of the *Streptomyces* natural product database StreptomeDB2.0^14^ uncovered 11 compounds with a mass range of 535–537 Da. Of these, only collinomycin from *Streptomyces collinus* was described as being red^15^. A comparison of the 16S rRNA gene sequence of the strains producing the 11 selected compounds revealed that the *S. collinus* sequence was the most similar to that of *Streptomyces* sp. no. 750 (98% identity). Therefore, we identified collinomycin as a candidate for sample A.

Additional data mining indicated that collinomycin had been renamed α-rubromycin (536.1 Da). To determine if our bioactive compound was α-rubromycin as opposed to the related compound β-rubromycin (536.4 Da), we compared ^1^H-NMR and ^13^C-NMR data of sample A versus α- and β-rubromycin standards. The rubromycins are distinguished by diverse oxidation states at C-3′, C-3, C-4 and the functionality at C-7^16^ (Supplementary Fig. S2). The sample A and β-rubromycin spectra were highly similar. The spectroscopic data for sample A were as follows: ^13^C-NMR (100 MHz) δ: 40.134 (C-3′), 29.605 (C-3), 22.162 (C-4), 52.928 (7-CO_2_CH_3_), 56.460 (5′-OCH_3_), and 57.118 (7′-OCH_3_). The spectroscopic data for β-rubromycin were as follows: ^13^C-NMR (100 MHz) δ: 40.134 (C-3′), 29.597 (C-3), 22.154 (C-4), 52.997 (7-CO_2_CH_3_), 56.452 (5′-OCH_3_), and 57.110 (7′-OCH_3_). Additionally, only sample A and β-rubromycin contained an 5,6-bisbenzannulated spiroketal, which distinguishes β-rubromycin from α-rubromycin^16^. Sample A also included three methoxy groups, which are signatures of β-rubromycin and are also used to differentiate the rubromycins. The ^1^H-NMR data for sample A and β-rubromycin were also highly similar (data not shown), although there were some differences in the ^1^H-NMR data as well as in the ^13^C-NMR between sample A and β-rubromycin due to contaminants. Furthermore, the UV/VIS spectral data indicated that the absorption maxima of sample A and β-rubromycin were 238 nm and 312 nm, respectively (Supplementary Fig. S3). These data suggested that sample A contained β-rubromycin and its derivatives.

**Figure 2:**
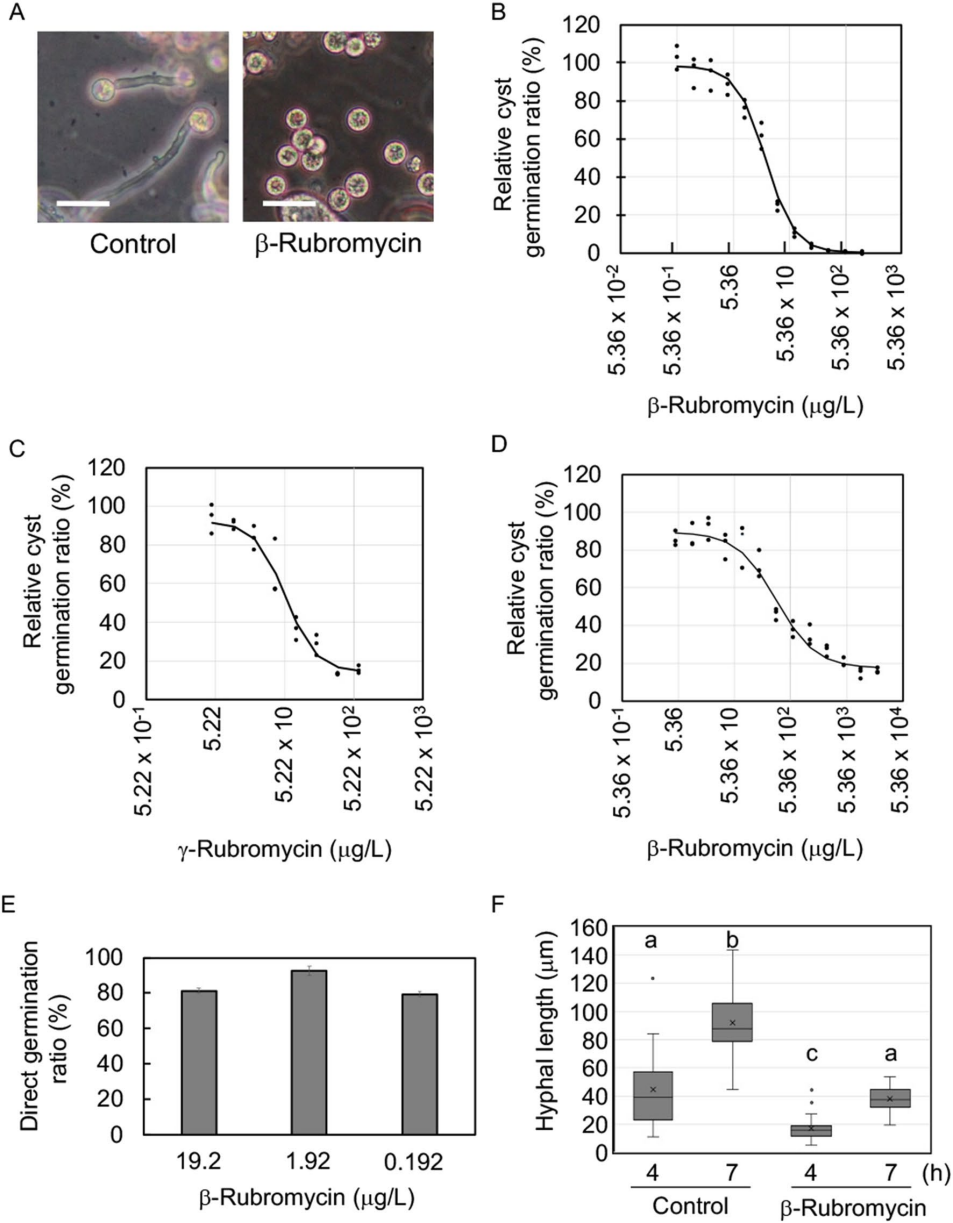
Effect of β-rubromycin and γ-rubromycin on *Ph. infestans* cyst germination or direct germination from sporangia. The indicated amount of each compound was added to sporangia or cysts, which were incubated at 10 °C for zoosporogenesis or 18 °C for direct germination for indicated time period and then scored (**A**–**D**). (**A**) Representative images of cysts with or without 1.0 mg/L β-rubromycin, showing the inhibition of cyst germination (at 6-h post stimulation of cyst germination). Bar is 50 μm. The indicated amount of β-rubromycin or γ-rubromycin was added to sporangia (**B** and **C**). Cyst germination was scored at 6-h after addition of a final concentration of 2.5 mM Ca(NO_3_)_2_ to zoospores. Comparison of the cyst germination rates in the presence of β-rubromycin (**B**) and γ-rubromycin (**C**). (**D**) The indicated amount of β-rubromycin was added to cysts just after adding a final concentration of 2.5 mM Ca(NO_3_)_2_ to zoospores, and which were incubated for 6 h at 18 °C and then cyst gemination was scored. (**E**) Effect of β-rubromycin on germination from sporangia. The indicated amount of β-rubromycin was added to sporangia and kept at 25 °C to assess the direct germination rate after 96 h. (**F**) Effect of β-rubromycin on hyphal elongation at 25 °C. The sporangium suspension was kept at 25 °C with or without 1.0 mg/L β-rubromycin. The length of hyphae was measured under a microscope at indicated time points. Letters indicate significant difference between groups (*p* < 0.05, one-way ANOVA). Each experiment was performed three times which contained three biological repeats.

To investigate the effects of commercially available rubromycins on *Ph. infestans* cyst germination, we purchased γ-rubromycin (molecular weight: 522.4) and compared its ability to inhibit cyst germination with purchased β-rubromycin, while α-rubromycin was not available commercially. *Ph. infestans* sporangia (1 × 10^3^ sporangia/40 μL) were mixed with various concentrations of the rubromycins in water. As mentioned earlier, the rubromycins did not prevent the release of zoospores. However, as illustrated in Fig. 2A, while germinated cysts were seen in the control no germination was observed in the presence of β-Rubromycin. A quantitative analysis by microscopy revealed that β-rubromycin inhibited cyst germination (IC_50_ = 19.8 μg/L) more effectively (Fig. 2B) than γ-rubromycin (IC_50_ = 58.5 μg/L), respectively (Fig. 2C). The difference between β-rubromycin and γ-rubromycin is the presence of a naphthazarin moiety in β-rubromycin, implying this group affects its effectiveness as a cyst germination inhibitor^16^. Addition of β-rubromycin to cysts also inhibited cyst germination (IC_50_ = 353.5 μg/L) (Fig. 2D). Zoospore release, cyst formation, and appressorium formation were unaffected by any of the compounds.

We next investigated whether β-rubromycin inhibits the direct germination of sporangia, which occurs at warmer conditions (e.g. 25 °C) than those used to produce zoospores. Suspensions comprising 1 × 10^3^ sporangia were treated with various β-rubromycin concentrations and then incubated at 25 °C for 96 h. β-Rubromycin did not inhibit the germination of sporangia, even at 19.2 mg/L (Fig. 2E). However, hyphal elongation delayed in the presence of 1.0 mg/L β-rubromycin at both 4 and 7-h after sporangia were kept at 25 °C (Fig. 2F). The delayed hyphal elongation was more obvious at 60 h (Supplementary Fig. S4). Thus, β-rubromycin attenuated hyphal elongation, but not sporangial germination.

### β-Rubromycin inhibited *Ph. infestans* infection of tomato leaves

**Figure 3:**
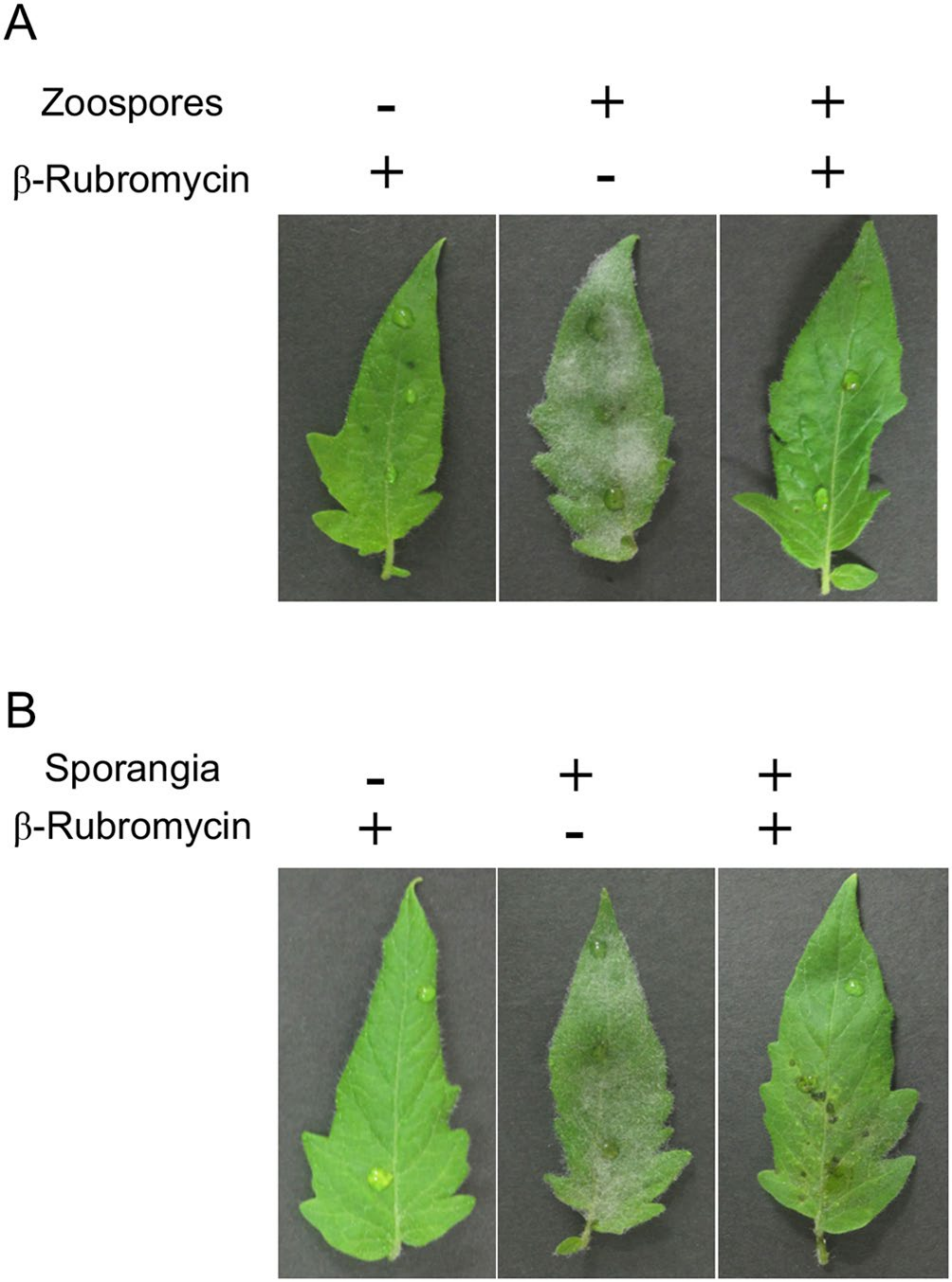
Effect of β-rubromycin on infection and *in planta* growth of *Ph. infestans*. (**A**) Tomato leaflets inoculated with zoospores with or without 1.0 mg/L β-rubromycin. The image was taken after 9 days. (**B**) Leaflets infected with a sporangium suspension with or without 1.0 mg/L β-rubromycin that was incubated at 25 °C for 8 h to stimulate direct germination. The image was taken after 9 days. Representative images for three biological replicates are presented.

We investigated whether the inhibition of cyst germination and hyphal elongation was correlated with decreased pathogenicity. To test the effect of the inhibitor on infection caused by zoospores, sporangia (1 × 10^4^ per mL) were incubated with or without 1.0 mg/L β-rubromycin at 10 °C for 2 h. More than 80% of the sporangia released zoospores under both conditions. Ten μl of zoospores (1 × 10^3^), purified from sporangia by passage through 15-μm pore mesh, were then added to each tomato leaflet. We observed that *Ph. infestans* colonized tomato leaves under the control condition, based on the presence of abundant surface hyphae and sporangia after 9 days at 18 °C (Fig. 3A). In contrast, no evidence of pathogen growth was observed when β-rubromycin had been added. We next investigated the effect of β-rubromycin on infection by directly germinated sporangia. Sporangia in the presence or absence of 1.0 mg/L β-rubromycin were incubated at 25 °C for 8 h to stimulate germination, after which time a 10-μL aliquot of the suspension was added to tomato leaflets. Hyphal growth of *Ph. infestans* was not observed in the presence of β-rubromycin (Fig. 3B). This implies that treating tomato tissue with β-rubromycin would also attenuate infection, although due to the cost of the compound this was not tested directly.

### β-Rubromycin inhibited germination of cysts and oospores in *Py. aphanidermatum*

**Figure 4:**
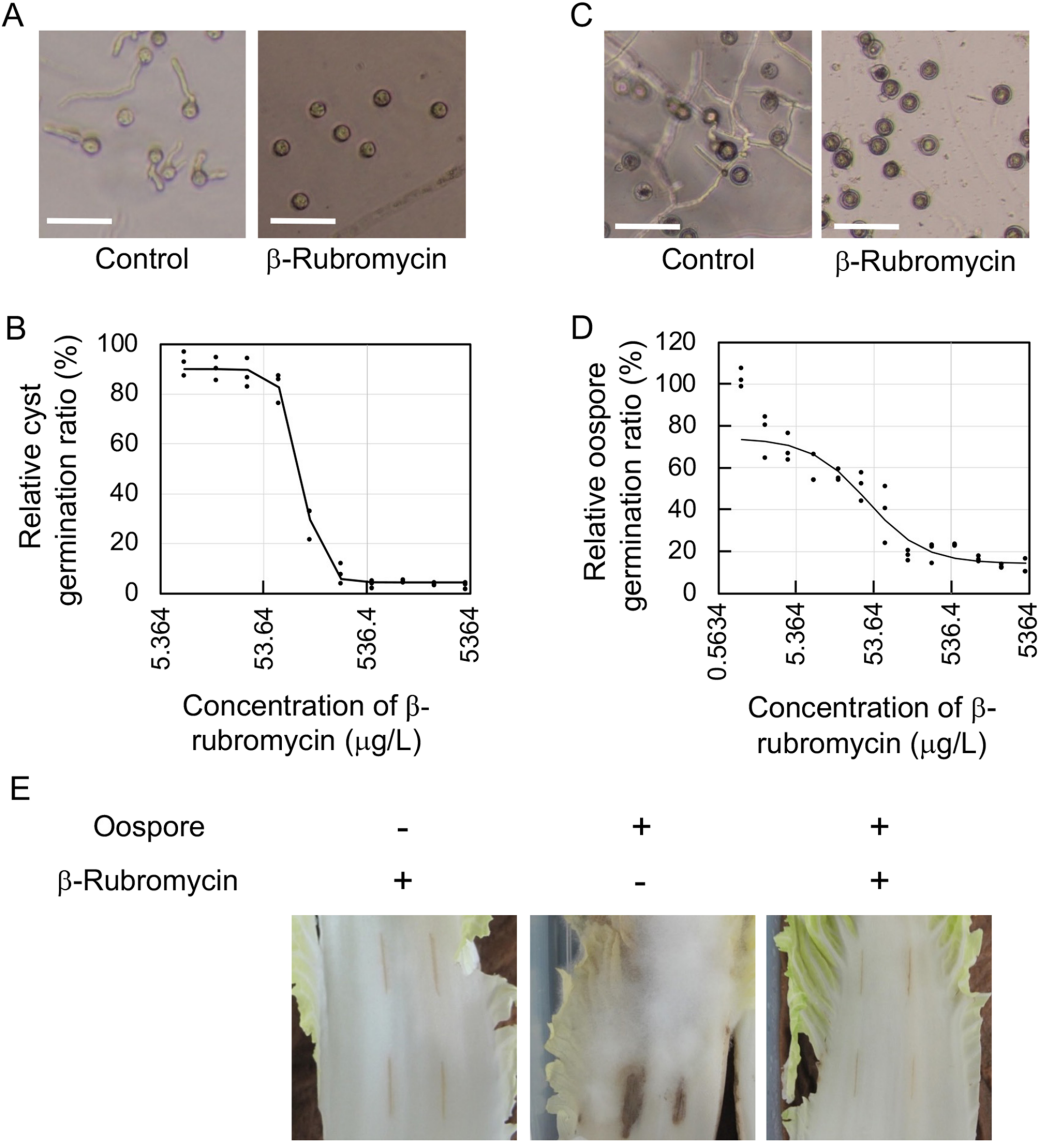
Effect of β-rubromycin on germination and *in planta* growth of *Py. aphanidermatum*. (**A**) Effect of 1.0 mg/L β-rubromycin on cyst germination. Representative data after 10 h at 18 °C are provided. Bar is 50 μM. (**B**) IC_50_ of β-rubromycin for cyst germination at 10 h after cyst germination was stimulated. Each experiment was performed three times which contained three biological repeats. (**C**) Effect of 1.0 mg/L β-rubromycin on oospore germination after 24 h. Representative data are provided. Bar is 50 μM. (**D**) IC_50_ of β-rubromycin for oospore germination after 24 h. Each experiment was performed three times which contained three biological repeats. (**E**) Effect of 1.0 mg/L β-rubromycin on *in planta* growth. Oospore (1.0 × 10^3^) suspensions with or without 1.0 mg/L β-rubromycin were added to wounded Chinese cabbage leaves (four straight cuts per leaf). Representative images for three biological replicates at 4 days post-inoculation are presented.

We next assessed whether β-rubromycin can inhibit cyst germination in *Ph. aphanidermatum*, causing *Pythium* damping-off. Since *Py. aphanidermatum* infections frequently involve its sexual cycle, we also examined whether β-rubromycin can inhibit germination of sexual spores (oospores) and its infection through oospores. β-Rubromycin significantly inhibited cyst germination (IC_50_ = 121.2 μg/L; Fig. 4A, B) as well as oospore germination (IC_50_ = 32.2 μg/L; Fig. 4C, D). Moreover, infection of Chinese cabbage was also inhibited by 1.0 mg/L β-rubromycin (Fig. 4E). These results demonstrated that β-rubromycin can be used as a reagent to study the mechanisms underlying morphological development in *Phytophthora* and *Pythium* species.

### Chemical genetic analysis to identify genes involved in morphological development in *Ph. infestans*

**Figure 5:**
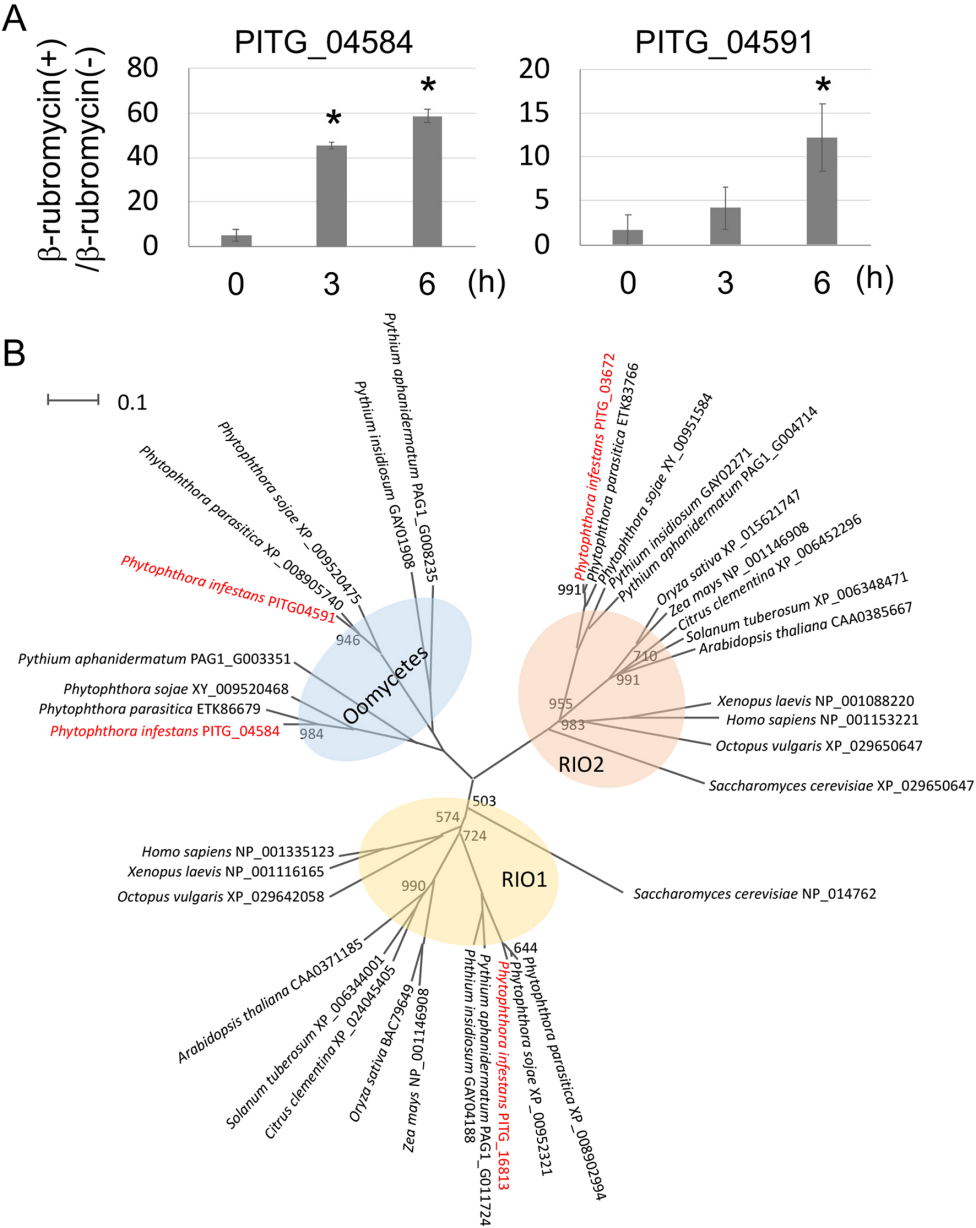
Expression and phylogenetic analyses of RIO kinase-like genes. (**A**) qRT-PCR of PITG_04584 and PITG_04591 expression in *Ph. infestans* with or without 1.0 mg/L β-rubromycin. Fold expression corresponds to the ratio of the mean expression levels of their genes in the presence of 1.0 mg/L β-rubromycin divided by those without β-rubromycin. The relative transcription levels are the means of at least three independent experiments, and the error bars indicate standard deviations. *p < 0.05, Student’s t-test. (**B**) The phylogenetic relationship of RIO kinase orthologs. Built is a consensus neighbor-joining tree based on sequences orthologous to PITG_04584, PITG_04591, PITG_03672, and PITG_16813, based on an alignment performed using ClustalW. The individual nodes of resulting trees was examined with 1000 bootstrap replicates; only values below 1000 are shown.

To obtain insight into the effect of β-rubromycin, we assessed gene expression profiles using RNA from cysts (0 h) and germinated cysts (3 and 6 h) treated with or without 1.0 mg/L β-rubromycin, respectively. Candidate genes of interest were identified by a preliminary RNA-seq experiment, and then validated by qRT-PCR, the latter involving three biological replicates. Since transcriptional profiles of many genes were modified in the presence of β-rubromycin, we first focused on checking the expression of transcription factor or kinase genes which were found in only oomycetes. One group of genes proved to show the most upregulated expression among putative transcription and kinase genes in qRT-PCR encoded two atypical protein kinases called RIO kinases. The addition of β-rubromycin caused these to be overexpressed by 60-fold (PITG_04584) and 12-fold (PITG_04591) in the 6-h germinated cysts (Fig. 5A). *Ph. infestans* possesses four RIO kinase-like genes (Fig. 5B). PITG_16813 possesses the STGKEA and IDxxQ signature sequences of RIO1 kinases, while PITG_03672 contains the GxGKES and IDFPQ signatures of the RIO2 group^17^. PITG_04591 possesses the SGKEA sequence which is one of two signature sequences of RIOB, SGKEA and IDxPQ^17^. The catalytic domain of the PITG_04584 is similar to those of RIO kinases although the above signature sequences were not conserved. A comparison of amino acid sequences using the FASTA algorithm revealed that PITG_04584 and PITG_04591 orthologs were found only in the genomes of oomycetes because their orthologs in plants and animals showed relatively higher e-value (approximately E−30 ~ E−35) and belonged to the clade of RIO2 and PITG_03672. In contrast PITG_16813 and PITG_03672 orthologs were found in a wide array of plants and other organisms with relatively lower e-value (< E−100) (Fig. 5B). Human RIOK1 (NP_001335123), yeast RIO1 (NP_014762), human RIOK2 (NP_029650647), and yeast RIO2 (XP_029650647), which were identified as non-ribosomal factors necessary for late 18 S rRNA processing^18–20^, were also included in these clades. Therefore, we first analyzed the function of an oomycete-specific gene, PITG_04584.

### PITG_04584 is involved in multiple steps of morphological development stimulated under cooler temperatures

**Figure 6:**
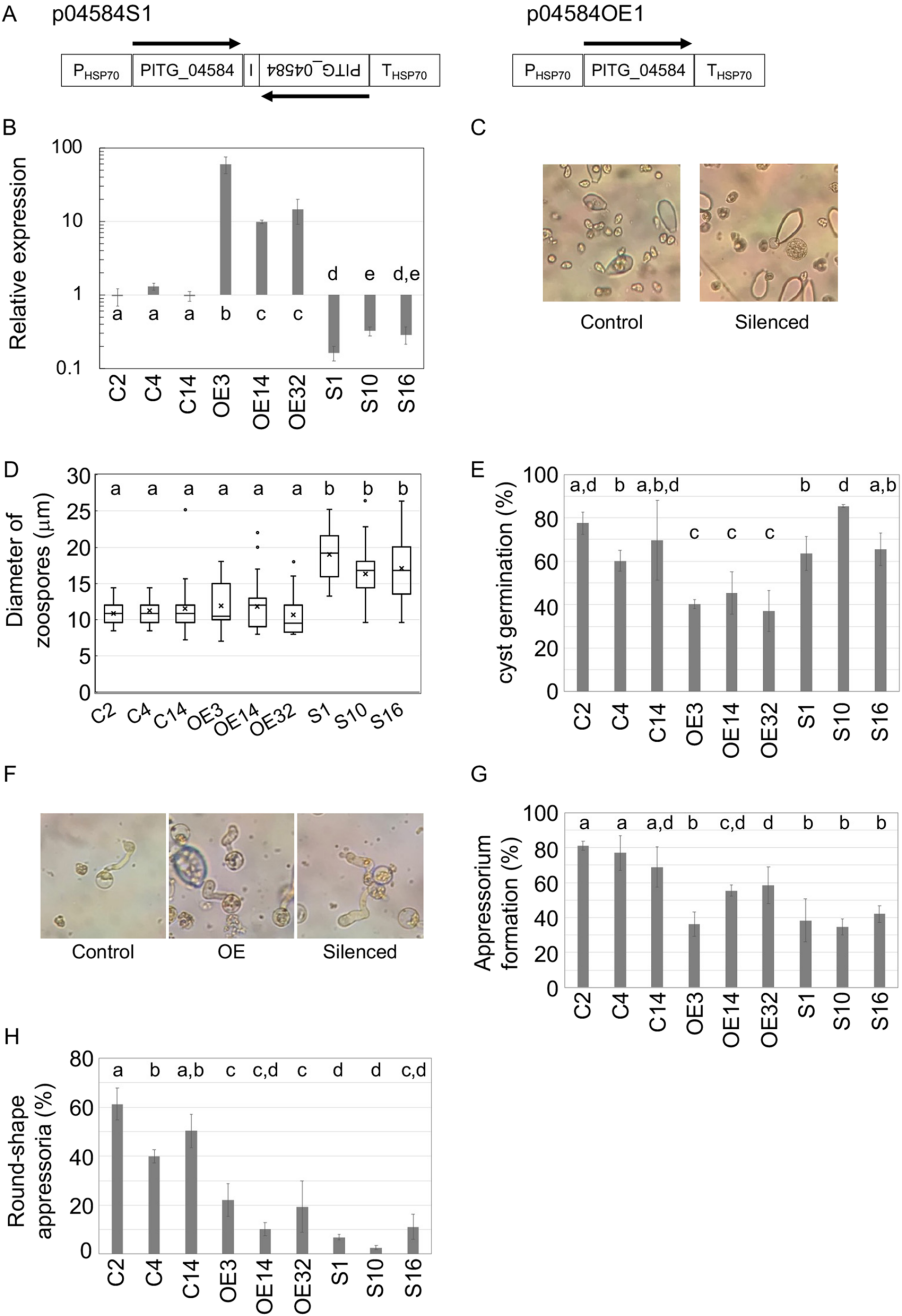
Genetic analysis of PITG_04584 function. (**A**) Plasmids used for silencing and overexpression. The maps are presented in linearized form. Hairpin constructs were expressed behind the constitutive HSP70 promoter (P_HSP70_), using sense and antisense PITG_04584 open reading frames (arrows) separated by the intron of the Ste20-like gene (I). The PITG_04584 gene was also overexpressed under the constitutive HSP70 promoter. The terminator of HSP70 (T_HSP70_) was used. Each plasmid also contains a *nptII* marker for selection. (**B**) qRT-PCR analysis of PITG_04584 in the transformants. Expression levels were normalized to a constitutive gene encoding ribosomal protein S3A. The relative transcript levels shown are the means of at least three independent experiments, and the error bars indicate the standard deviations. Letters indicate significant differences between groups (*p* < 0.05, one-way ANOVA). (**C**) Representative pictures of zoospores in the control (C2) and silenced strains (S1). (**D**) The diameter of zoospores were measured from three different areas under the microscope (n > 30 in each). Results from three independent experiments are depicted as the mean ± s.d. Letters indicate significant differences between groups (*p* < 0.05, one-way ANOVA followed by Tukey’s post-test). (**E**) Cyst germination rates, calculated by dividing the number of germinated cysts by that of the sum of ungerminated and germinated cysts. Letters indicate significant differences between groups (*p* < 0.05, one-way ANOVA). (**F**) Representative pictures of appressoria in control C2, overexpressing strain OE3, and silenced mutant S1. (**G**) Fraction of germinated cysts producing appressoria. (**H**) Fraction of appressoria showing an oval shape, defined as those having a height/width ratio > 1.5. Results from three independent experiments are depicted as the mean ± s.d. Different letters indicate significant differences between groups (*p* < 0.05, one-way ANOVA). Each experiment was performed three times which contained three biological repeats.

Gene overexpression and homology-based gene silencing were used to help assess the role of the PITG_04584 gene on morphological development. PITG_04584 was expressed 60 times higher compared to empty vector control (C2, C4, and C14) in transformant OE3, 10 times in OE14, and 12 times in OE32, and reduced to one-sixth of the controls in silenced transformant S1, one-third in S10, and one-third in S14 (Fig. 6B). Since neighbors within 500 nt of the target gene are often cosilenced^21^, we investigated the expression of PITG_04583, which resides within 540 nt of PITG_04584; there was no predicted gene on the other side of PITG_04584 within 40 kb. Since the expression of PITG_04583 in all transformants was not significantly different from wild-type (Supplementary Fig. S5), we investigated phenotypes resulting from silencing and over-expression. Hyphal elongation and sporangium formation on rye agar media and zoospore release at 10 °C were not affected (Supplementary Fig. S6A–C). However, the average diameter of zoospores was 16–19 μm in the three silenced mutants compared to 10–12 μm in the three control and overexpressing strains; this represents a significant difference (*p* < 0.01) (Fig. 6C, D). Although cyst formation rates were almost the same in each strain, cyst germination rates were significantly reduced in three overexpressing mutants to two-thirds that of the three control and silenced strains (*p* < 0.05) (Fig. 6E). It should be noted that the reduction of cyst germination in the overexpressing strains is consistent with the inhibitory effect of β-rubromycin on that developmental transition and the increased expression of PITG_04584 in the presence of β-rubromycin. In addition, appressorium formation was significantly reduced in both the overexpressing and silenced mutants (*p* < 0.05) (Fig. 6G). Furthermore, shape of appressoria differed in both the silenced and over-expressing strains. While the height/width ratio of wild-type appressorium is < 1.5, appressoria were more elongated in the two classes of transformants (Fig. 6F, H). Despite the defects in formation of normal zoospores in the silenced strains, in cyst germination in the overexpressing mutants, and in appressorium formation in both the overexpressing and silenced strains, each successfully infected tomato leaves (data not shown).

## Discussion

In this study, we first identified β-rubromycin as a *Ph. infestans* cyst germination inhibitor by screening compounds produced by *Streptomyces* isolated from soil*.* β-Rubromycin inhibited *Ph. infestans* cyst germination and hyphal elongation from sporangia, while not affecting zoospore release, cyst formation, or appressorium formation. Furthermore, the compound inhibited the germination of *Py. aphanidermatum* cysts and oospores. Chemical genetic analyses using β-rubromycin identified a RIO kinase-like gene, PITG_04584, as a critical contributor to zoosporogenesis, cyst germination, and the formation of appressoria in *Ph. infestans*.

The effects of rubromycins on viruses, bacteria, and human cancer cells have been thoroughly investigated^16^. Previous studies showed that β-rubromycin, γ-rubromycin, and 3′-hydroxy-β-rubromycin inhibit the growth of *Bacillus subtilis*, *Staphylococcus aureus*, and *Escherichia coli.* In eukaryotes, rubromycins are inhibitory toward viral reverse transcriptase and human telomerase^22^. Comparative analyses of rubromycins revealed that all examined moieties (i.e., quinone, spiroketal, and isocoumarin) are required for telomerase inhibition^23^. These results are consistent with our observations indicating that the naphthazarin moiety is critical for the inhibition of *Ph. infestans* cyst germination (Fig. 2).

In humans, a previous study proved that β-rubromycin competitively interacts with the telomerase substrate primer, TS-A (*K*_i_ = 0.74 μM), enabling β-rubromycin to interact with the telomerase RNA^22^. Although β-rubromycin showed nonspecific cytotoxicities, it inhibited the proliferation of both K-562 and Hela cells with IC_50_ values of 19.5 μM and 22.7 μM, respectively^21^. While oomycetes do contain orthologs of human telomerase, it is possible that β-rubromycin affects other targets since it had no effect on vegetative growth on rye medium. Also, the IC_50_ for cyst germination (37 nM) was much lower than that observed for the inhibition of human cells.

Calcium signaling plays a central role in zoosporogenesis^10,24^. One study revealed that approximately two-thirds of the genes activated during zoosporogenesis rely on calcium signaling^11^. However, the molecular mechanisms underlying development remain relatively unknown in oomycetes. A few factors are reportedly involved in cyst germination. For example, the silencing of a gene encoding a dynamin-related protein (*PsVPS1*) was reported to result in abnormal cyst germination and inhibited infections of soybean by *Phytophthora sojae*^25^. In *Phytophthora capsici*, leucine-rich repeat (LRR) domain-containing kinases contribute to sporangium formation, zoospore release, cyst germination, and infection of *Nicotiana benthamiana*^26^. Our preliminary analysis of transcription in the presence of β-rubromycin during cyst germination indicated that the expression levels of genes encoding dynamin-like proteins (PITG_08836, 08837, and 08838) and an LRR receptor kinase gene (PITG_17495) were unaffected by β-rubromycin (data not shown).

A striking finding of our study is that RIO kinase-like gene, PITG_04584, was required for normal zoospore development. RIO kinases, which possess limited sequence homology with canonical eukaryotic protein kinases, include four subfamilies: RIO1, RIO2, RIO3, and RIOB^17,27^. RIOB is found in some eubacteria where its function remains to be determined^27^. In contrast, RIO1 and RIO2 are highly conserved from archaea to human and have been assigned functions based largely on studies of human and yeast. Both kinases in yeast and human are involved in the late stages of 18 S rRNA processing^18–20^. RIO1 in yeast is also involved in entry into S phase and exit from mitosis^28^. RIO3 is present only in metazoans, where it is required for normal processing of 21 S pre-rRNA^29^ and regulation of the NF-κB signaling pathway through its interaction with caspase-10^30^. Based on phylogenetic analysis PITG_16813 and PITG_03672 might play roles for ribosomal RNA processing. It has been revealed that number of RIO kinase paralogs vary between taxa and their functions may vary. Baker et al. proposed that paralog interference is a common constraint on the evolution of gene duplicates including their resolution, which can generate additional regulatory complexity^31^. The PITG_04584 group of RIO kinases, which occurs throughout the oomycete group, may therefore have evolved to have a novel function in oomycetes following an ancient gene duplication event.

The inhibitory effect of β-rubromycin on cyst germination was consistent with the expression profile of PITG_04584 and the phenotypes of its mutants, although we have not identified the target of β-rubromycin. So far, ATP-competitive inhibitors such as toyocamycin and a series of pyridine caffeic acid benzyl amides are known as inhibitors of RIO kinases^32,33^. RIO kinases contain a canonical eukaryotic protein structure, but also display several unusual structural features; implying that the identification of the target proteins of PITG_04584 and the inhibitor of PITG_04584 may contribute to establish new methods to suppress diseases caused by oomycetes.

## Materials and methods

### Manipulation of *Ph. infestans* and *Py. aphanidermatum*

*Phytophthora infestans* isolate 1306 is an A1 strain that was isolated from tomato in California, USA. Importation of this isolate was approved by the Ministry of Agriculture, Forestry, and Fisheries of Japan. The culture was maintained at 18 °C on rye agar^34^. Sporangia were released from 10-day cultures by adding water, rubbing the surface of the medium with a glass rod, and eliminating hyphal fragments by passage through 50-μm nylon mesh. The collected sporangia (2 × 10^4^ per ml) were either incubated at 10 °C for zoosporogenesis or at 25 °C for direct germination. Zoospores were obtained and purified by incubating the sporangia for 120 min followed by passage through 15-μm mesh. Cysts were obtained by adding Ca(NO_3_)_2_ to 2.5 mM followed by incubation for 30 min at 18 °C. Germinating cysts were obtained by adding one fiftieth volume of clarified rye media to the cyst suspension followed by incubation for 6 h at 18 °C. Appressorium formation was scored 8-h after addition of the clarified rye media to the cysts. Aliquots were removed at the times noted in Results and viewed under a microscope (Olympus CKX41 inverted microscope, Tokyo, Japan) to assess morphological change, basing measurements on a minimum of 100 cells. Each experiment was performed three times which contained three biological repeats. More than 80% of sporangia typically released zoospores, more than 80% of cysts germinated, and more than 80% of germinated cysts formed appressoria under a control condition. For RNA analysis, cysts and germinating cysts were pelleted at 1000 × *g* for 5 min at 4 °C, and then frozen in liquid nitrogen.

*Py. aphanidermatum* isolate OPU854^35^ was maintained on V8 juice agar at 25 °C^36^. Zoospore release and cyst formation in *Py. aphanidermatum* were stimulated as described previously^37^. We collected *Py. aphanidermatum* oospores from 5-d mycelia as described^38^, after which the oospore suspensions were adjusted to 1 × 10^3^ oospores/mL.

### Screening microorganisms for compounds affecting cyst germination

Actinomycetes were isolated from soil samples using modified HVG agar medium as described^39^. The HVG was modified by addition of Pridham-Godleave solution, which contains trace elements and 1.5% (w/v) gellan gum (Supplementary Table S1). Isolated microorganisms were cultured on maltose-Bennett’s agar. An acetone extract was prepared from cultures grown for 5-d in liquid medium A at 30 °C (Supplementary Table S1) by adding an equal volume of acetone followed by mixing. For bioassays, 20-μL aliquots were mixed with 1 × 10^3^ *Ph. infestans* sporangia in total 70 μL (14.2% acetone solution), incubated at 10 °C for 18 h, and examined using an inverted microscope (Olympus, Tokyo, Japan). As a control, we confirmed that 15% acetone had no effect on morphological change in *Ph. infestans*.

Isolated microorganisms were identified based on 16S rRNA analysis. This involved polymerase chain reaction (using primers 5′-AGAGTTTGATCCTGGCTCAG and 5′-AAGGAGGTGATCCAGCCGCA^40^) followed by Sanger sequencing.

### Optimization of fermentation conditions

Isolates strains were initially cultured in medium A at 30 °C for 2 d. Aliquots were then transferred to 200 mL of the six media described in Supplementary Table S1 and incubated at 30 °C for 6 d. EtOAc extracts were prepared daily and assayed for their abilities to inhibit cyst germination.

### Isolation of the cyst germination inhibitor

*Streptomyces* sp. no. 750 was cultured in liquid medium E (Supplementary Table S1) at 30 °C for 5 days. The supernatant (76.4 L) was extracted twice with EtOAc followed by evaporation. The crude extract (10.76 g) was added to a Wakogel C-200 column (FUJIFILM Wako Pure Chemical Corporation, Osaka, Japan) and eluted with a stepwise gradient of EtOAc/MeOH (100:0 to 0:100) to produce two fractions (EtOAc/MeOH, 80:20, 70:30) that yielded 0.77 g dry material. This was resuspended in MeCN and purified by HPLC with an Inertsil ODS-3 column (GL Sciences, Tokyo, Japan). The gradient elution was as follows: H_2_O/MeCN (90:10) for 10 min, H_2_O/MeCN (from 90:10 to 0:100) for 20 min, and H_2_O/MeCN (0:100) for 10 min. The flow rate was set at 2.0 mL/min and the eluent was fractionated every 1 min. The cyst germination inhibitor was detected in the 16th fraction (30 mg). The bioactive compound was further purified by HPLC with the Inertsil ODS-3 column and an isocratic elution involving 50% MeCN. Only a single peak was detected, which was dried to yield 2.9 mg of a red powder.

### Spectroscopic analysis of the purified compound and β-rubromycin

The structures of the purified compound (sample A) and β-rubromycin (AdipoGen Life Sciences, San Diego, CA) were determined based on ^1^H- and ^13^C-NMR. This used DMSO-*d*_6_ with a JNM AL-400 NMR spectrometer (JEOL Ltd. Tokyo, Japan). Chemical shifts were determined using the solvent peak (δ_H_ 2.49, δ_C_ 39.7) as an internal standard. Molecular mass was determined with the LC–MS 2020 system (Shimadzu).

### Bioassays using purified sample A and rubromycins

The sample A and commercially purchased rubromycins, β-rubromycin and γ-rubromycin (AdipoGen Life Sciences), were dissolved in DMSO. Assays of *Ph. infestans* were conducted as described above using 2 μL of inhibitor at the concentrations described in Results with 1 × 10^3^ sporangia (in 98 μL) or 5 × 10^3^ cysts (in 98 μL). IC_50_ values for rubromycins were determined based on a linear regression.

Assays involving *Py. aphanidermatum* were performed as follows. An oospore suspension (approximately 1 × 10^3^ in 178 μL) was mixed with 2 μL β-rubromycin at the amounts indicated in Results and 20 μL 5% V8 liquid medium. The resulting mixture was incubated at 25 °C for 30 min. We then added Ca(NO_3_)_2_ to a final concentration of 2.5 mM, followed by incubation for 24 h to stimulate germination. Oospore morphology was examined with a microscope. Approximately 1 cm^2^ V8 plugs containing *Py. aphanidermatum* were dipped in 10 mL sterilized water at room temperature to stimulate zoospore release. The water was refreshed 1 h later, and zoospore release observed with a microscope. Approximately 10^4^ zoospores were used to assess the effect of β-rubromycin on cyst germination. Aliquots were removed at the times noted in Results and viewed under an inverted microscope to assess morphological change, basing measurements on a minimum of 100 cells. Each experiment was performed three times which contained three biological repeats.

### Infection assays

Plant infection assays were conducted with the leaves of tomato plants (cv. Momotaro) grown for 3–5 weeks at room temperature. Tomato leaflets inoculated with *Ph. infestans* sporangium or zoospore suspensions were incubated at 18 °C for 7–9 days under humid conditions. Infection assays were performed with a 10-μL aliquot from a 98-μL sporangium suspension (3–5 × 10^3^/mL) and a 98-μL zoospore suspension (3–5 × 10^3^/mL) with or without 2 μL various concentration of β-rubromycin in DMSO. Three droplets were spotted to each leaflet. Three leaflets were used per replicate. Each experiment was performed three times which contained three biological repeats.

A second infection assay was completed with Chinese cabbage leaves infected with *P. aphanidermatum*. Specifically, an oospore suspension was prepared as described above. The oospore suspension (1 × 10^3^ in 198 μL) supplemented with 2 μL β-rubromycin was used to inoculate Chinese cabbage leaves, which were then incubated at 35 °C under humid conditions for 4 days. Each experiment was performed three times.

### Transformation of *Ph. infestans*

Transformations were conducted by using the electroporation method and G418 selection^41^, using circular plasmids p04584S1 and p04584OE1 (shown Fig. 6A without the *nptII* marker gene). Plasmids p04584OS1 and p04584OE1 were constructed in pHAM35 by inserting the HSP70 promoter (P_HSP70_) from pSTORA. In pRIOS1, open reading frames from PITG_04584 in sense and antisense orientations separated by an 85-nt intron from the Ste20-like gene^42^ were expressed under control of P_HSP70_. In p04584OE1, the open reading frame from PITG_04584 was inserted into the vector in sense orientation to express the gene under the control of P_HSP70_. Primers used for plasmid construction are listed in Supplementary Table S2.

### Quantitative real-time RT-PCR

qRT-PCR was performed using SYBR Green detection as described previously^43^. In brief, primers for qRT-PCR of PITG_04584 (Supplementary Table S2) were designed to amplify the 3′-UTR of the target gene. Assays were based on a minimum of three biological replicates using three technical replicates per tissue sample. Control amplifications were performed using no reverse transcriptase, and melt curves confirmed the fidelity of the amplification. Expression levels were calculated using the ΔΔC_T_ method, using a constitutive gene (ribosomal protein S3A, PITG_11766) as a control^44^.

### RNA-sequencing analysis

This was performed as described^45^. In brief, libraries were prepared using the Illumina TruSeq RNA Sample Prep Kit v2 according to standard protocols (Illumina, San Diego, CA, USA). Each RNA sample (1 μg) was enriched for mRNA using oligo (dT)-tagged beads. The mean insert size for each library was approximately 280 to 300 bp. Sequencing was performed in a paired-end 50 base mode on a Miseq system (Illumina). We prepared only one sample for each condition: cysts (time 0), germinated cysts (3 and 6 h) with or without 1 mg/mL β-rubromycin. The sequences were analyzed using the CLC genomics workbench (CLC Bio, Aarhus, Denmark). Only reads with quality values higher than Q30 were used for mapping. These were mapped to *Ph. infestans* genome data from fungiDB (https://fungidb.org/fungidb/app/search/dataset/AllDatasets/result) to calculate RPKM values.

## Supplementary Information

Supplementary Information.

## Data Availability

All data generated or analyzed during this study are included in this published article and its Supplementary Information Files.
