# Supplementary material for: Chemical genetic approach using β-rubromycin reveals that a RIO kinase-like protein is involved in morphological development in Phytophthora infestans
Source: Sci Rep. 2020 Dec 18;10:22326. doi: 10.1038/s41598-020-79326-7 (PMC7749174; doi:10.1038/s41598-020-79326-7)

Supplementary to manuscript: Chemical genetic approach using β-rubromycin reveals that a RIO kinase-like protein is involved in morphological development in *Phytophthora infestans*

Shuji Tani*[1], Naotaka Nishio[1], Kenji Kai[1], Daisuke Hagiwara[2,3], Yoshiyuki Ogata[1], Motoaki Tojo[1], Jun-ichi Sumitani[1], Howard S. Judelson[4], Takashi Kawaguchi[1]

Supplementary Fig. S1. Dose-dependent inhibition of *P. infestans* cyst germination by the acetone extract sample from the isolated microorganism. The cyst germination rate under the control condition (none, no extract sample) was set as 100%. The extract sample was sequentially diluted with sterilized water and added to sporangia. The presented representative data are based on the results of two biological replicates.

Supplementary Fig. S2. The structure of α-rubromycin, β-rubromycin, and γ-rubromycin.

Supplementary Fig. S3. UV/VIS spectral data for sample A (a) and β-rubromycin (b).

Supplementary Fig. S4. Effect of β-rubromycin on hyphal elongation at 25°C. The sporangium suspension was kept at 25°C with or without 1.0 mg/L β-rubromycin. (A) The length of hyphae was measured with a microscope at indicated time points. The mean (closed circle, control condition; open circle, 1mg/L β-rubromycin) and standard deviation (bars) of the hyphal length at indicated time points. (B) Representative images of sporangia treated with or without 1.0 mg/L β-rubromycin. Bar is 50 μm. ND means not determined. Each experiment was performed three times which contained three biological repeats.

Supplementary Fig. S5. qRT-PCR analysis of PITG_04583 expression in each strain. Expression levels were calculated using the $\Delta\Delta C_T$ method, using a constitutive gene (ribosomal protein S3A, PITG_11766) as a control. The relative transcription levels are the means of at least three independent experiments, and the error bars indicate the standard deviations. There were no significant difference among the strains.

Supplementary Fig. S6. Morphological development of each strain. (A) Hyphal growth of each strain on rye media. Hyphal length from the inoculated point. There

were no significant difference among the strains. (B) Sporangia were released from 10-day-old cultures and counted at more than three independent replicates. There were no significant difference among the strains. (C) The collected sporangia were incubated at 10°C for zoospore release. There were no significant difference among the strains.

Supplementary Table S1. Composition of media used to optimize β-rubromycin production in *Streptomyces* sp. #750

	A	B	C	D	E	F
Glucose	1.0%	1.0%	-	1.0%	1.0%	1.0%
Dextrin	2.0%	-	2.0%	-	2.0%	-
Starch	-	2.0	-	-	-	-
Glycerol	-	-	1.0	-	-	-
Bacto Soytone	1.5%	1.5%	1.5%	-	1.5%	-
Soybean flour	-	-	-	1.0%	-	1.0%
Yeast Extract	0.1%	0.1%	0.1%	-	0.1%	-
$CaCO_3$	0.3%	0.3%	0.3%	0.1%	0.3%	0.1%
NaCl	-	-	-	0.5%	-	0.5%
*Pridham-Godleave solution	-	-	-	-	1 mL/L	1 mL/L
pH	7.0	7.0	7.0	7.0	7.0	7.0

*Pridham-Godleave solution

$FeSO_4 \cdot 7H_2O$	1.1 g/L
$MnCl_2 \cdot H_2O$	7.9 g/L
$ZnSO_4 \cdot 7H_2O$	1.5 g/L
$CuSO_4 \cdot 5H_2O$	06.4 g/L

Supplementary Table S2. Primers used in this study.

Name	Sequence (5' to 3')
For the construction of p04584S1	
Hsp70F	AAGAGCTCTATGACCATGATTACGCCAA
Hsp70R	AACCGCGGTCCATCTTGTTCAATCATGC
04584senF	AAGCGGCCGCATGTTCGACGAGGACACACA
04584senR	TACCCTTCTCTCAGTTACGTAGCAGCGGCT
Ste20intF	ACGTAACTGAGAGAAGGGTAAGCCAAGACA
Ste20intR	AAACTAGTTAGGACCTAAAGAATCGCCA
04584antiF	AACCCGGGATGTTCGACGAGGACACACA
04584antiR	AAACTAGTTCAGTTACGTAGCAGCGGCT
For the construction of p04584OE1	
04584_OEF	AAACTAGTATGTTCGACGAGGACACACA
04584_OER	AACCCGGGTCGCGTTATTCCAAAAGG
For quantitative RT-PCR	
Q04584F	CGTGGAGTGGACTTCACTGG
Q04584R	CGCTCACTCTCCGTCCTACA
Q04591F	GGTCATGCAGTTTGTGGGAG
Q04591R	TGCAGGATGTCAGCGTATGT
Q04583F	GGACCTGGTTGCTGAGTGTC
Q04583R	TGTGCACCTCAAACTTGGTC

Under lines indicate the overlapping region for fusion PCR.

Supplementary Fig. S1

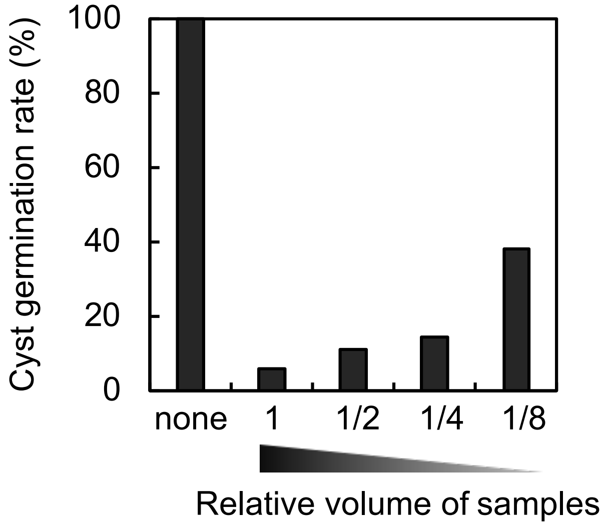

Supplementary Fig. S2

α-rubromycin

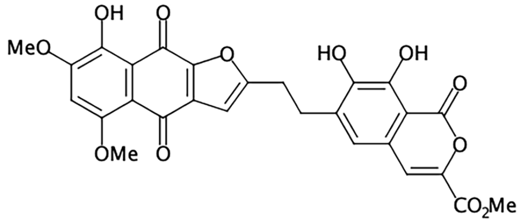

β-rubromycin

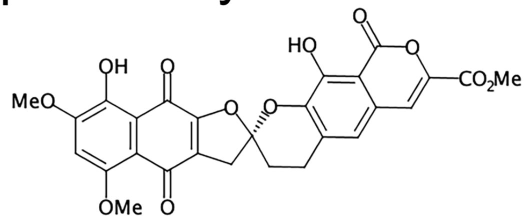

γ-rubromycin

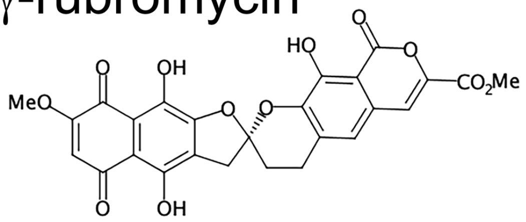

Supplementary Fig. S3

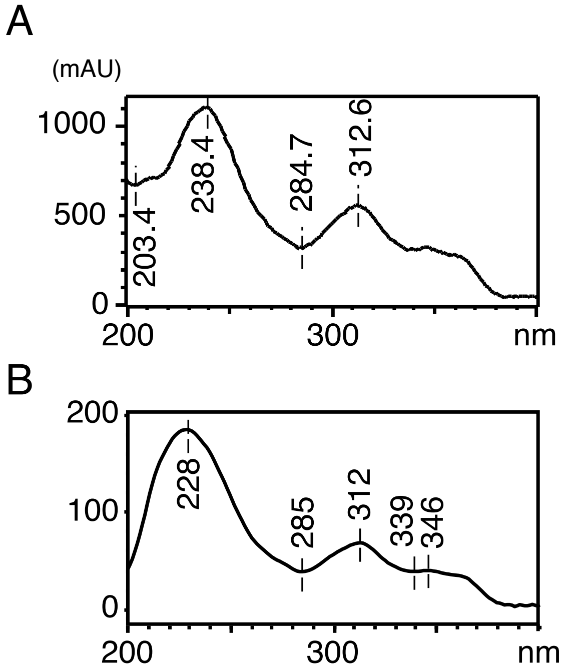

Supplementary Fig. S4

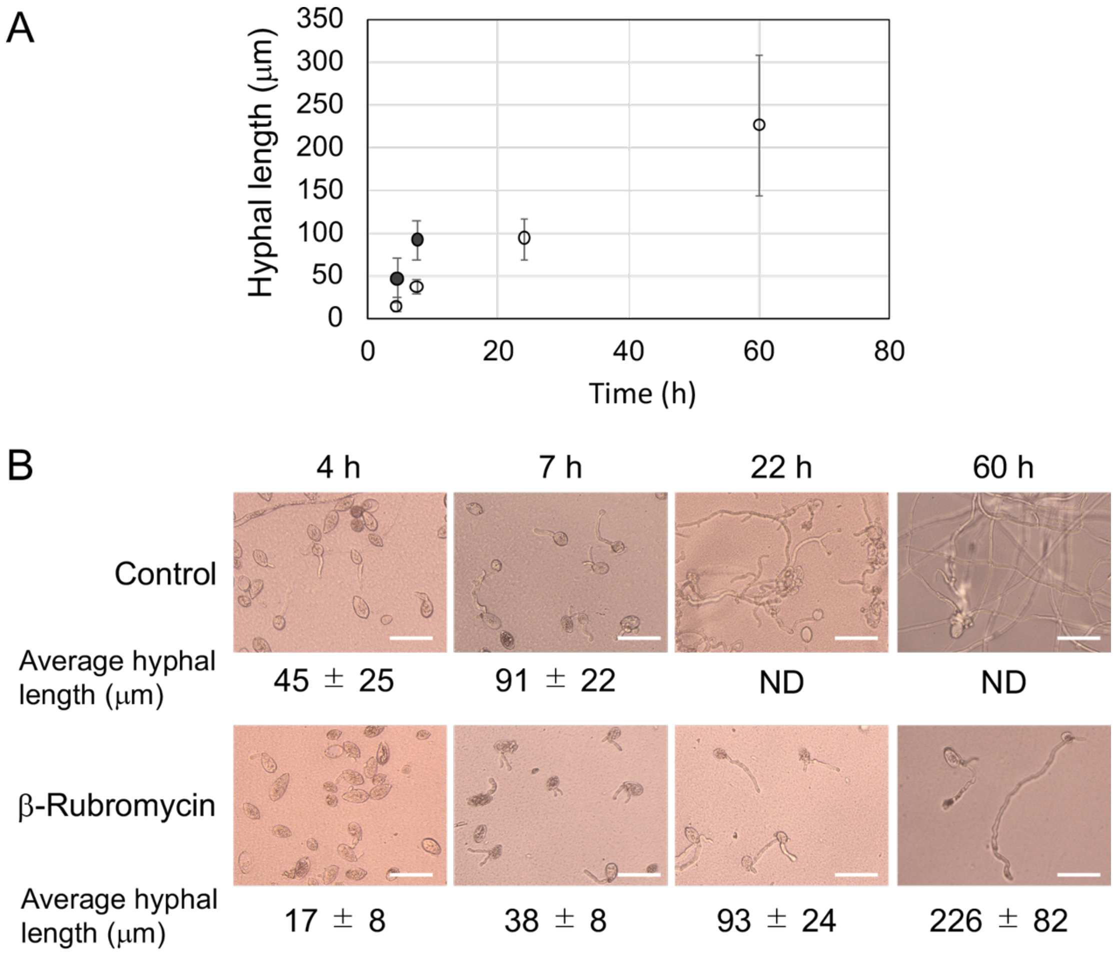

Supplementary Fig. S5

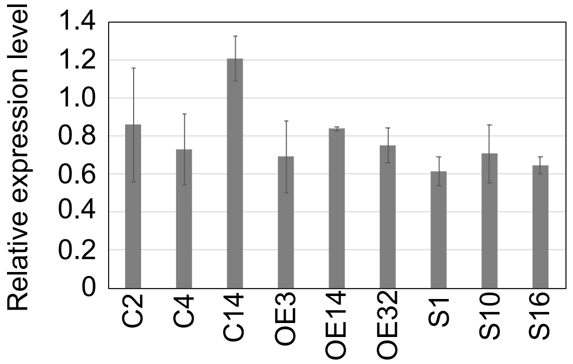

Supplementary Fig. S6

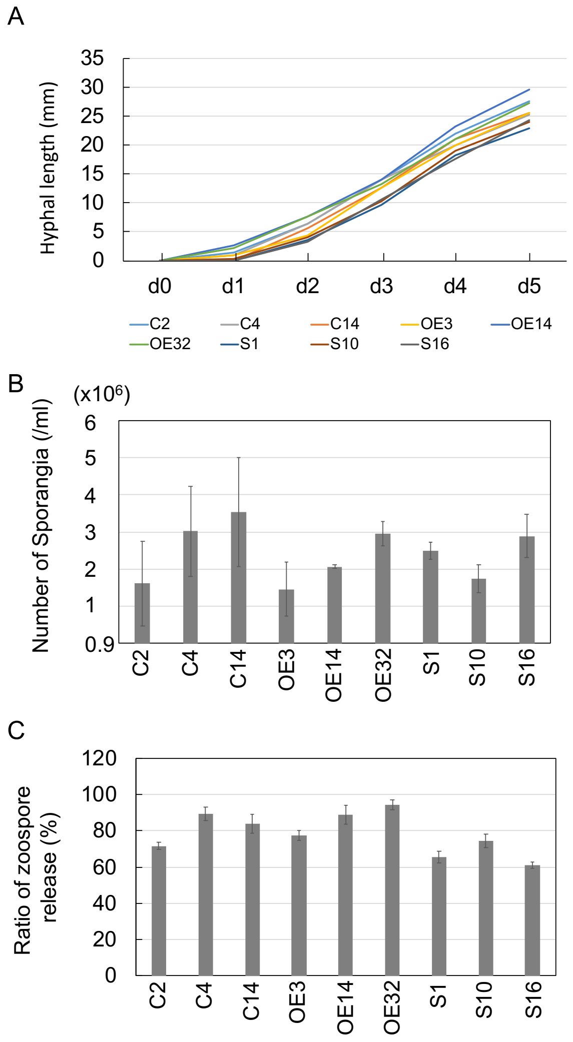

Supplement: Supplementary file 1 — Supplementary Information. [file 41598_2020_79326_MOESM1_ESM.pdf]
